# PCR identification of toxic euglenid species *Euglena sanguinea*

**DOI:** 10.1007/s10811-017-1376-z

**Published:** 2018-01-09

**Authors:** Agata Kulczycka, Maja Łukomska-Kowalczyk, Bożena Zakryś, Rafał Milanowski

**Affiliations:** Department of Molecular Phylogenetics and Evolution, Institute of Botany, Faculty of Biology, Biological and Chemical Research Center, University of Warsaw, ul. Żwirki i Wigury 101, 02-089 Warsaw, Poland

**Keywords:** *Euglena sanguinea*, Euglenophycin, Toxic blooms, DNA barcoding, Molecular identification

## Abstract

*Euglena sanguinea* Ehrenberg is the only known species of euglenids which forms toxic blooms causing tangible losses to fish farms. *Euglena sanguinea* produces euglenophycin, a toxin similar in structure to solenopsin, an alkaloid found in fire ant venom. It was proved that euglenophycin exhibits not only ichthyotoxic but also herbicidal and anticancer activity. Recently, a specific mass spectrometric method of identification and quantitation of euglenophycin was developed to facilitate monitoring of that toxin in freshwater ponds. Despite the recent taxonomic verifications, proper identification of *E. sanguinea* is still difficult, especially for less experienced researchers. Herein, we describe a simple method based on nested PCR amplification of the nSSU rDNA fragments to identify a single *E. sanguinea* cell and its detection in a sample of water. The method will further facilitate monitoring of water reservoirs, especially estimating the risk of toxic blooms.

**Electronic supplementary material:**

The online version of this article (10.1007/s10811-017-1376-z) contains supplementary material, which is available to authorized users.

## Introduction

At the beginning of the twenty-first century, the occurrence of toxic algae blooms in freshwater aquaculture ponds was reported 13 times in the USA (North and South Carolina, Texas, Arkansas, and Mississippi). Lost revenue from these events exceeded US$1.1 million (Zimba et al. 2004, 2010). The dominant algae species was a euglenid (Excavata, Euglenozoa, Euglenida), which was isolated, cultured, and recognized as the fish mortality-inducing factor. The euglenid species present in toxic blooms was identified as *Euglena sanguinea*. The toxicity was observed both for isolates taken from the infested ponds as well as for the clonal strain from the culture collection. The toxin produced by *E. sanguinea*, called euglenophycin, was identified and described. It is an alkaloid similar in structure to fire ant venom, solenopsin. It was proved that euglenophycin exhibits not only ichthyotoxic but also herbicidal and anticancer activity (Zimba et al. 2010, 2016). Recently, a specific mass spectrometric method of identification and quantitation of euglenophycin was developed to facilitate monitoring of that toxin in freshwater ponds (Gutierrez et al. 2013). The latest laboratory experiments revealed that euglenophycin is produced not only by *E*. *sanguinea* but also by seven other euglenid species: *Euglena sociabilis* P. A. Dangeard, *Euglena stellata* Mainx, *Euglenaria clavata* (Skuja) Karnkowska-Ishikawa & E.W. Linton, *Euglenaria anabaena* (Mainx) Karnkowska-Ishikawa & E.W. Linton, *Strombomonas borystehnienis* (Y. V. Roll) T. G. Popova, *Trachelomonas ellipsoidalis* K. P. Singh, and *Lepocinclis acus* (O. F. Müll.) B. Marin & Melkonian (Zimba et al. 2017). However, *E. sanguinea* remains the only known species of euglenids to form toxic blooms.

*Euglena sanguinea* is a cosmopolitan species which can be found in shallow, calm, and eutrophic freshwater systems. It was one of the first green euglenid species described in the literature (Ehrenberg 1831). However, its correct identification was problematic due to complicated chloroplast morphology—the original diagnostic feature (Pringsheim 1956). In effect, during over 200 years of studying euglenids, 12 new taxa resembling *E*. *sanguinea* were named, although their correct identification based on morphology alone was practically impossible. Recently, a review of the description of *E*. *sanguinea* and species similar to it was conducted by verifying morphological and molecular data. The result of the analysis was a reduction of the number of species from 12 to four (*E*. *sanguinea*, *E*. *sociabilis*, *Euglena splendens* P. A. Dangeard, and *Euglena laciniata* E. G. Pringsheim). Furthermore, new epitypes and updated diagnostic descriptions were also established for them (Karnkowska-Ishikawa et al. 2013). Finally, the most significant diagnostic features were recognized: the presence of fusiform mucocysts, the number of chloroplasts, the size of the double-sheathed pyrenoids, and the presence of the large paramylon grain in the vicinity of the stigma. However, despite taxonomic verifications, proper identification of this species is still challenging, particularly for less experienced researchers. The method allowing unambiguous recognition of *E*. *sanguinea* is based on the use of its nSSU rDNA as a molecular barcode.

DNA barcoding is a powerful method for species-level identification (Hajibabaei et al. 2007), particularly for inexperienced researchers. It is fast, accurate, and does not require morphological analyses (Blaxter 2004). There is no universal barcode—several markers, such as COI, ITS, nSSU rDNA, *matK*, and *rbcL*, are used for different eukaryotic organisms. For phototrophic euglenids, the variable regions V2–V3 and V4 of nSSU rDNA seem to be the best barcodes (Łukomska-Kowalczyk et al. 2016). Unfortunately, *E*. *sanguinea* is the sole species of the group for which the use of standard methods of nSSU rDNA amplification has proved unsatisfactory. The reason is the unusual structure of this sequence, which is much longer than in any other species. The length of the sequence from the strain SAG 1224-30 is over 6000 bp and seems to be the longest known SSU rDNA sequence (Karnkowska-Ishikawa et al. 2013). The amplification of very long variable regions V2–V3 and V4 is far from efficient and molecular identification of *E*. *sanguinea* using standard methods is problematic. Therefore, we decided to refine the species-specific PCR test, which enables recognition of *E*. *sanguinea* through the peculiarity of its nSSU rDNA. This method can further facilitate monitoring of freshwater ponds and estimating the risk of toxic blooms formed by *E*. *sanguinea*.

## Materials and methods

### Strains, culture conditions, and environmental samples

Five strains of *Euglena sanguinea* from algae collections were used in the study: SAG 1224-30 (SAG, Sammlung von Algenkulturen, Pflanzenphysiologisches Institut der Universität Göttingen, Germany, as *Euglena magnifica* E.G.Pringsheim), Henderson (the strain isolated from toxic bloom from a pond in Texas), MI-20 and MI-51 (MI, Michigan isolate Triemer Lab, Department of Plant Biology, Michigan State University), and ACOI 1267 (ACOI, Culture Collection of Algae at the Department of Botany, University of Coimbra, Portugal). In the control group experiments, DNA samples from nine *Euglena* species were used: *E*. *rubra* A. D. Hardy (MI 103), *E*. *splendens* (MI 47), *E*. *tristella* S. P. Chu (NJ, New Jersey isolate Triemer Lab, Department of Plant Biology Michigan State University), *E. sociabilis* (ACOI 920), *E*. *deses* Ehrenberg (ASW08075, now available from CCAC, Culture Collection of Algae, University of Cologne, Germany as *E*. *intermedia* Matvienko CCAC 2443 B), *E*. *clara* Skuja (SAG 25.98), *E*. *gracilis* G. A. Klebs (SAG 1224-5/25), *E*. *laciniata* (SAG 1224-31), and *E*. *viridis* Ehrenberg (SAG 1224-17d). All strains were cultivated in a liquid soil–water medium enriched with a small piece of garden pea (medium 3c, Schlösser 1994) and kept in a growth chamber maintained at 17 °C and a 16:8-h light/dark cycle, ca. 27 μmol photons m^−2^ s^−1^ provided by cool white fluorescent tubes. Additionally, three environmental fresh water samples from Poland containing *E*. *sanguinea* cells were used. Environmental sample 1 was collected from a small pond in Rudawka village (53° 51′ 56.5″ N, 23° 30′ 52.6″ E) in July 2015; the other two samples were collected from field ponds near Urwitałt village: sample 2 (53° 49′ 09.5″ N, 21° 39′ 21.8″ E) in June 2011 and sample 3 (53° 50′ 43.1″ N, 21° 36′ 42.3″ E) in June 2012. From each pond, a 10-L sample was collected, and plankton nets with a mesh size of 10, 50, and 100 μm were used to increase density (up to 1 L) and exclude bigger plankton organisms and other macroscopic objects. Samples were transported to the laboratory and were centrifuged (100 mL of each sample); the sediment was suspended in 10 mL of water and split into separate Eppendorf tubes (1 mL) and stored at − 20 °C until needed for DNA isolation. The presence of *E*. *sanguinea* cells in samples was confirmed with a NIKON Eclipse E-600 microscope with a differential interference contrast, equipped with the NIS-Elements Br 3.1 software (Nikon). Also, the population density of each species was estimated as follows: (o) cells very occasionally observed in one drop (50 μL of the 10-mL sample after centrifugation), (+) 5–10 cells, (++) 11–20 cells, (+++) 21–30 cells, and (++++) over 30 cells.

### Primer designing

Based on the alignment of all available euglenid nSSU rDNA sequences, the regions for primer design were chosen according to the following principles: (i) regions conserved for *E*. *sanguinea* (GenBank numbers: strain Argentina JQ281804, Henderson JQ281805, and SAG 1224-30 JQ281806), but dissimilar to any other species of euglenids, were chosen; (ii) intraspecific variations within the region were flanked by the primers; and (iii) the length of the PCR product had to be appropriate for efficient amplification. Two sets of species-specific primers were designed manually—the external primers sangF0/R0 (encompassing the region between helix 29 and 45 in the secondary structure of nSSU rDNA; sangF0: CTGYGGGCGCCACGCCCCCTTG, sangR0: ACGGACTTGCRGGGTTTCCCAGC) and the internal primers sangF1/R1 (between helix 30 and 45; sangF1: CGCCCCCTTGACCGAGAAATCCG, sangR1: GCCRGGGCCCRCAGAARACGAGG).

### PCR templates

Three types of templates were used: (i) DNA isolated from cultures, (ii) DNA from lysis of a single cell/a defined number of cells, and (iii) DNA isolated from environmental samples (fresh water reservoirs). Total genomic DNA from cell cultures and environmental samples had been purified with DNeasy Tissue Kit (Qiagen) in accordance with the animal tissues protocol. Single cell lysis was performed according to the Lax and Simpson (2013) procedure, slightly modified. Single cells were isolated with a micropipette using a micromanipulator (MM-89, Narishiege) installed on a Nikon Ni-U microscope and collected in 0.2-mL PCR tubes. Probes with 1, 5, 30, and 100 cells of *E*. *sanguinea* were prepared. Liquid traces were removed by centrifuging in a Speed Vac concentrator, followed by the addition of 5 μL of the Phusion GC PCR buffer (no additional buffer was used in the subsequent PCR reaction). The cells were lysed using five freeze/thaw cycles (liquid nitrogen/heating block 95 °C) and used directly in PCR.

### PCR amplification and sequencing

The annealing temperature for the two sets of primers was optimized independently in a gradient PCR reaction (50–72 °C). The final conditions were as follows: a 25-μL reaction mixture contained 0.5 U Phusion High-Fidelity DNA Polymerase (Thermo Scientific), 0.2 mM dNTPs, 1.5 mM MgCl_**2**_, 5 pmol of each primer, reaction buffer GC (Thermo Scientific), and Q-solution (Qiagen). The PCR protocol consisted of 2 min at 98 °C, followed by nine initial cycles comprising the following steps: 30 s at 98 °C, 30 s at 62 (sangF0/R0) or 60 °C (sangF1/R1), and 20 s at 72 **°**C, then by 39 cycles comprising steps of 15 s at 98 °C, 15 s at 62 or 60 °C, and 20 s at 72 °C. The final extension step was performed for 5 min at 72 **°**C. As a template, 10–50 ng of DNA was used in standard PCR reaction, but low concentrations of DNA were also tested in the range 1–0.001 pg. Nested PCR was used in order to make the reaction more sensitive and specific (sangF0/R0 primers in the first round, sangF1/R1 in the second round), particularly for amplification of DNA derived from single/defined number of cells. The conditions were as described above. In the second round as a template, 1 μL of the mixture from the first round was used. The PCR protocol for the first round was as described above (annealing 62 °C); the second round consisted of the initial step 2 min at 98 °C, followed by 39 cycles comprising 15 s at 98 °C, 15 s at 60 °C, and 20 s at 72 °C. The final extension step was performed for 5 min at 72 °C. The control PCR reactions were also performed with DNA stemming from various *Euglena* species. All PCR reactions were carried out in the presence of positive (DNA from *E*. *sanguinea*) and negative (water or buffer) controls. Chosen PCR products were sized on agarose gels, purified and sequenced directly from both strands using the BigDye Terminator Cycle Sequencing Ready Reaction Kit 3.1 (Applied Biosystems).

## Results

**Fig. 1:**
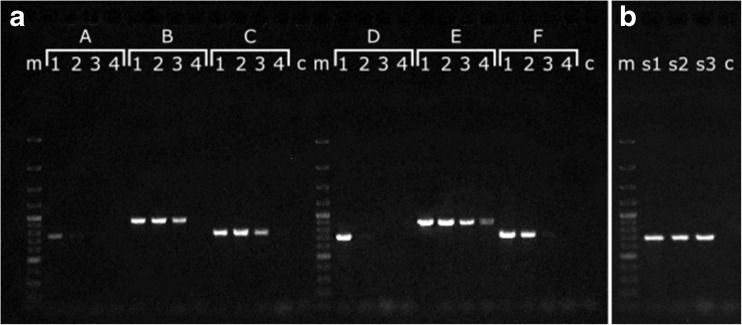
**a** Agarose gel electrophoresis of PCR products from a dilution series of template DNA from three strains of *E*. *sanguinea* (A, D) Henderson, (B, E) SAG 1224-30, and (C, F) MI-51; (A–C) amplified with the sangF0/sangR0 primer pair; (D–F) amplified with the sangF1/sangR1 primer pair; lanes (1–4) PCR products from 1, 0.1, 0.01, and 0.001 pg of template DNA, respectively; (m) GeneRuler 100 bp Plus DNA ladder; (c) negative control. **b** Agarose gel electrophoresis of PCR products for DNA isolated form environmental fresh water samples with confirmed presence of *E*. *sanguinea* cells; (s1) sample; (s2) sample 2; (s3) sample 3; (m) GeneRuler 100 bp Plus DNA lader; (c) negative control

Designed primer pairs amplified efficiently and specifically the fragment of nSSU rDNA from *E*. *sanguinea* strains (length of PCR products with sangF0/R0: SAG 1224-30—921 bp, Henderson, MI-20 and ACOI 1267—743 bp; sangF1/R1: SAG 1224-30—878 bp, Henderson, MI-20 and ACOI 1267—700 bp, MI-51—717 bp, GenBank no. KY928280) in a wide range of annealing temperatures (54–64 °C for sangF0/R0 and 50–66 °C for sangF1/R1). The optimal temperature for the pair of external primers sangF0/R0 was 62 °C. For internal primers sangF1/R1, the optimal temperature was 60 °C. The obtained sequences of PCR products were identical for the strains Henderson, MI-20, and ACOI 1267. Therefore, they were considered as genetically indistinguishable and the latter two strains were not included in subsequent analyses. The test for a minimal amount of template in PCR reactions revealed that 1 pg of DNA is enough for efficient amplification in the case of the three examined strains of *E*. *sanguinea*. The reaction proved to be the most sensitive for strain SAG 1224-30—even the amount of 0.01 pg of DNA resulted in PCR products of good quality (Fig. 1a). The specificity of primers used in reactions was also tested. Single PCR reactions with primers sangF0/R0 and sangF1/R1, as well as nested amplification, gave negative results for nine *Euglena* species, including *E*. *rubra*, *E*. *splendens*, *E*. *laciniata*, and *E*. *sociabilis*, the species most closely related to *E*. *sanguinea*. In turn, nested PCR tests enabled efficient amplification of the selected nSSU rDNA region even from single cells of *E*. *sanguinea*. This test gave positive results for 1–30 cells lysed by the freeze/thaw method. However, using cell numbers greater than 30 led to a decrease in efficiency, most likely due to the higher concentration of PCR inhibitors in unpurified samples. The nested PCR test was also used successfully for detection of *E*. *sanguinea* in three environmental probes (Fig. 1b), where its presence along with other species of euglenids (representing genera *Discoplastis*, *Euglena*, *Euglenaria*, *Euglenaformis*, *Lepocinclis*, *Monomorphina*, *Phacus*, *Strombomonas*, and *Trachelomonas*) had been confirmed earlier by microscopic analysis (supplementary tables S1–3, supplementary material online). In the first environmental probe (sample 1), besides *E*. *sanguinea*, *E*. *splendens* was equally abundant; both species are closely related and morphologically very similar (supplementary table S1, supplementary material online). In sample 2, no euglenids closely related or morphologically similar to *E*. *sanguinea* were present (supplementary table S2, supplementary material online). In sample 3, *E*. *sanguinea* and *E*. *splendens* coexisted together, but this time the *E*. *splendens* population was significantly larger (supplementary table S3, supplementary material online). Sequencing of PCR products confirmed genetic similarity of *E*. *sanguinea* in environmental samples to the strain Henderson.

## Discussion

Euglenids have been researched for almost 200 years now. During that time, more than 3000 species have been described (3200 validly published names listed in Algaebase: http://www.algaebase.org) and intensive research regarding the biochemistry, molecular biology, and phylogeny of euglenids has been carried out (Zakryś et al. 2017). For this reason, it is surprising that the toxicity of *E*. *sanguinea*, which can cause measurable damage to the economy, has been severely overlooked. Furthermore, recent studies have shown that euglenid species other than *E*. *sanguinea* produce euglenophycin (*E. stellata*, *E*. *sociabilis*, *Eu. anabaena*, *Eu*. *clavata*, *L. acus*, *T. ellipsoidalis*, *S. borystehnienis*; Zimba et al. 2017). The analysis included 33 species and seven different isolates of *E*. *sanguinea*. Interestingly, it was revealed that the species capable of producing toxins are not closely related but occur in different branches of the phylogenetic tree. On this basis, it can be expected that many other species of euglenids that were not included in the analysis can also produce and accumulate euglenophycin. The toxicity of some euglenids certainly plays an important role in their functioning in specific ecological niches. This, however, is of little importance to the economy. The situation differs in the case of *E*. *sanguinea*, as its dense blooms constitute a real threat to aquaculture, and thus to human population.

As mentioned in “Introduction,” using classic microscopic methods for identification of *E*. *sanguinea* is challenging, even for experts from the field (Karnkowska-Ishikawa et al. 2013). Therefore, the ability to identify the species based on molecular data seems to be very useful. The effectiveness of such identification has been demonstrated both in laboratory tests with breeding strains and environmental samples. It has been shown that a very small amount of DNA template gives positive result even in one-rounded PCR reaction. The test enabled also efficient amplification of target region in the strain of *E*. *sanguinea* (MI-51) which nSSU rDNA sequence was not previously published and was not used in primer designing. It leads to a conclusion that developed test would work properly also for other *E*. *sanguinea* strains which are currently unknown. Moreover, the nucleotide sequence of obtained PCR products brings additional information, allowing for assignment of examined sample to previously described strain or its qualification as the new one. On the other hand, no products were observed in PCR reactions for the closest relatives of *E*. *sanguinea*, which proves specificity of the test. The analysis of environmental samples, in which *E*. *sanguinea* was present at various densities and accompanied by many closer or further related eugenid taxa (supplementary tables S1–3, supplementary material online), gave similar results as for breeding strains. Particular attention should be paid to sample 3 in which the presence of *E*. *sanguinea* was detected despite a very low abundance. Such result suggests that developed method may be a very useful tool for monitoring water reservoirs in terms of the presence of *E*. *sanguinea* cells.

At present, PCR-based methods are commonly used to detect and identify a variety of organisms, including toxic algae (Galluzzi et al. 2007). The test presented herein also utilizes this methodology and allows for analysis of *E*. *sanguinea* at various levels: (1) the detection of *E*. *sanguinea* in a sample of water taken from the environment, (2) identification of individual cells, and (3) sequences of obtained PCR products can be used as barcodes allowing for estimation of intraspecific genetic variation and comparison of particular isolates to examined strains, including those of confirmed toxicity. This assay, together with a specific mass spectrometric method of identification and quantitation of euglenophycin (Gutierrez et al. 2013), will further facilitate monitoring of water reservoirs, particularly estimation of the risk of *E*. *sanguinea* toxic blooms.

## Electronic supplementary material

ESM 1 (DOCX 15.4 kb)

ESM 2 (DOCX 15.0 kb)

ESM 3 (DOCX 15.1 kb)

## References

[CR1] Blaxter ML (2004). The promise of a DNA taxonomy. Philos Trans R Soc B.

[CR2] Ehrenberg CG (1831) Über die Entwickelung und Lebensdauer der Infusionsthiere; nebst ferneren Beiträgen zu einer Vergleichung ihrer organischen Systeme. Abhandlungen der Königlichen Akademie der Wissenschaften Berlin. 1831:1–154; Tafeln I–IV

[CR3] Galluzzi L, Magnani M, Saunders N, Harms C, Bruce IJ (2007). Current molecular techniques for the detection of microbial pathogens. Sci Prog.

[CR4] Gutierrez DB, Rafalski A, Beauchesne K, Moeller PD, Triemer RE, Zimba PV (2013). Quantitative mass spectrometric analysis and post-extraction stability assessment of the euglenoid toxin euglenophycin. Toxins.

[CR5] Hajibabaei M, Singer GA, Hebert PD, Hickey DA (2007). DNA barcoding: how it complements taxonomy, molecular phylogenetics and population genetics. Trends Genet.

[CR6] Karnkowska-Ishikawa A, Milanowski R, Triemer RE, Zakryś B (2013). A redescription of morphologically similar species from the genus *Euglena*: *E*. *laciniata*, *E*. *sanguinea*, *E*. *sociabilis*, and *E*. *splendens*. J Phycol.

[CR7] Lax G, Simpson AG (2013). Combining molecular data with classical morphology for uncultured phagotrophic euglenids (Excavata): a single-cell approach. J Eukaryot Microbiol.

[CR8] Łukomska-Kowalczyk M, Karnkowska A, Krupska M, Milanowski R, Zakryś B (2016). DNA barcoding in autotrophic euglenids: evaluation of COI and 18S rDNA. J Phycol.

[CR9] Pringsheim EG (1956). Contributions towards a monograph of the genus *Euglena*. Nova Acta Leopoldina.

[CR10] Schlösser UG (1994) SAG-Sammlung von Algenkulturen at University of Göttingen. Catalogue of Strains 1994. Bot Acta 107:111–186

[CR11] Zakryś B, Milanowski R, Karnkowska A, Schwartzbach S, Shigeoka S (2017). Evolutionary origin of *Euglena*. *Euglena*: biochemistry, cell and molecular biology.

[CR12] Zimba PV, Rowan M, Triemer RE (2004). Identification of euglenoid algae that produce ichthyotoxin(s). J Fish Dis.

[CR13] Zimba PV, Moeller PD, Beauchesne K, Lane HE, Triemer RE (2010). Identification of euglenophycin-a toxin found in certain euglenoids. Toxicon.

[CR14] Zimba PV, Ordner P, Gutierrez DB (2016). Selective toxicity and angiogenic inhibition by euglenophycin: a role in cancer therapy?. J Cancer Biol Treat.

[CR15] Zimba PV, Huang IS, Gutierrez D, Shin W, Bennett MS, Triemer RE (2017). Euglenophycin is produced in at least six species of euglenoid algae and six of seven strains of *Euglena sanguinea*. Harmful Algae.

