# Supplementary material for: PCR identification of toxic euglenid species Euglena sanguinea
Source: J Appl Phycol. 2018 Jan 9;30(3):1759–63. doi: 10.1007/s10811-017-1376-z (PMC5982438; doi:10.1007/s10811-017-1376-z)
Supplement: Supplementary file 1 — (DOCX 15.4 kb) [file 10811_2017_1376_MOESM1_ESM.docx]

**S 1.** Species composition in the environmental sample 1 collected from a small pond in Rudawka village (53°51'56.5"N 23°30'52.6"E) in July 2015. The population density (observed in 50 µl of the 10 ml sample after centrifugation) was estimated as follows: (o) cells very occasionally observed, (+) 5-10 cells, (++) 11-20 cells, (+++) 21-30 cells, (++++) over 30 cells.

| Species name | Population density |
| --- | --- |
| *Discoplastis spathirhyncha* (Skuja) Triemer | o |
| *Euglena ehrenbergii*  G. A. Klebs | ++++ |
| ***Euglena sanguinea* Ehrenberg** | **++** |
| *Euglena splendens* P. A. Dangeard | ++ |
| *Lepocinclis ovum* var. *gracillicauda* Deflandre | ++ |
| *Lepocinclis horridus* (Pochm.) M. S. Bennet & Triemer | ++ |
| *Lepocinclis ovum* var. *fominii* Roll | + |
| *Lepocinclis tripteris* (Dujardin) B. Marin & Melkonian | + |
| *Lepocinclis acus* (O.F. Müll.) B. Marin & Melkonian | o |
| *Lepocinclis fusiformis* (H. J.Carter) Lemmermann | o |
| *Lepocinclis steinii* Lemmermann | o |
| *Phacus hamatus* Pochmann | + |
| *Phacus helicoides* Pochmann | + |
| *Phacus caudatus* Hübner | + |
| *Phacus rotunda* (Pochmann) Zakryś & M. Łukomska | o |
| *Phacus salina* (Fritsch) E. W. Linton & A. Karnkowska-Ishikawa | o |
