# Supplementary material for: PCR identification of toxic euglenid species Euglena sanguinea
Source: J Appl Phycol. 2018 Jan 9;30(3):1759–63. doi: 10.1007/s10811-017-1376-z (PMC5982438; doi:10.1007/s10811-017-1376-z)
Supplement: Supplementary file 2 — (DOCX 15.0 kb) [file 10811_2017_1376_MOESM2_ESM.docx]

**S 2.** Species composition in the environmental sample 2 collected from a small pond near Urwitałt village (53°49'09.5"N 21°39'21.8"E) in June 2011. The population density (observed in 50 µl of the 10 ml sample after centrifugation) was estimated as follows: (o) cells very occasionally observed, (+) 5-10 cells, (++) 11-20 cells, (+++) 21-30 cells, (++++) over 30 cells.

| Species name | Population density |
| --- | --- |
| ***Euglena sanguinea* Ehrenberg** | **+++** |
| *Euglena tristella* S.P. Chu | + |
| *Euglena ehrenbergii* G. A. Klebs | + |
| *Euglena chadefaudii* Bourrelly | o |
| *Euglenaria clavata* (Skuja) Karnkowska-Ishikawa & E.W. Linton | ++++ |
| *Euglenaria clepsydroides* Zakryś | ++ |
| *Euglenaformis proxima* (P. A. Dang.) M.S. Bennett & Triemer | ++++ |
| *Monomorphina aenigmatica* (Drezep.) Nudelman & Triemer | + |
| *Monomorphina pyrum* (Ehrenb.) Mereschkowsky | o |
| *Lepocinclis fusca* (G.A. Klebs) Kosmala & Zakryś | o |
| *Lepocinclis ovum* (Ehrenb.) Minkiewicz | o |
| *Lepocinclis oxyuris* (Schmarda) B. Marin & Melkonian | o |
| *Phacus arnoldii* Swirenko | + |
| *Phacus anomalus* F. E. Fritsch & M. F. Rich | + |
| *Phacus lismorensis* Playfair | + |
| *Phacus longicauda* (Ehrenb.) Dujardin | + |
| *Phacus orbicularis* Hübner | + |
| *Phacus acuminatus* A. Stokes | o |
| *Trachelomonas volvocina* Ehrenberg | ++++ |
| *Trachelomonas hispida* (Perty) F. Stein | +++ |
| *Trachelomonas volvocinopsis* Svirenko | +++ |
