# Supplementary material for: PCR identification of toxic euglenid species Euglena sanguinea
Source: J Appl Phycol. 2018 Jan 9;30(3):1759–63. doi: 10.1007/s10811-017-1376-z (PMC5982438; doi:10.1007/s10811-017-1376-z)
Supplement: Supplementary file 3 — (DOCX 15.1 kb) [file 10811_2017_1376_MOESM3_ESM.docx]

**S 3.** Species composition in the environmental sample 3 collected from a small pond near Urwitałt village (53°50'43.1"N 21°36'42.3"E) in June 2012. The population density (observed in 50 µl of the 10 ml sample after centrifugation) was estimated as follows: (o) cells very occasionally observed, (+) 5-10 cells, (++) 11-20 cells, (+++) 21-30 cells, (++++) over 30 cells.

| Species name | Population density |
| --- | --- |
| *Euglena tristella* S. P. Chu | +++ |
| *Euglena splendens* P. A. Dangeard | + |
| *Euglena deses* Ehrenberg | o |
| *Euglena ehrenbergii* G. A. Klebs | o |
| ***Euglena sanguinea* Ehrenberg** | **o** |
| *Euglenaformis proxima* (P. A. Dang.) M. S. Bennett & Triemer | o |
| *Lepocinclis ovum* (Ehrenb.) Minkiewicz | ++ |
| *Lepocinclis acus* (O. F. Müll.) B. Marin & Melkonian | o |
| *Lepocinclis fusca* (G.A. Klebs) Kosmala & Zakryś | o |
| *Phacus lismorensis* Playfair | o |
| *Phacus arnoldii* Svirenko | o |
| *Phacus anomalus* F. E. Fritsch & M. F. Rich | o |
| *Phacus hamatus* Pochmann | o |
| *Phacus salina* (Fritsch) E. W. Linton & A. Karnkowska-Ishikawa | o |
| *Strombomonas urceolata* (Stokes) Deflandre | o |
| *Trachelomonas armata* (Ehrenb.) F. Stein | ++++ |
| *Trachelomonas abrupta* Svirenko | +++ |
| *Trachelomonas superba* Svirenko | ++ |
| *Trachelomonas volvocinopsis* Svirenko | ++ |
| *Trachelomonas volvocina* Ehrenberg | + |
